# 17,18-Dibromo-8-methyl-4,12-ditosyl-3,4,5,6,7,8,9,10,11,12,13,14-dodeca­hydro-2*H*-benzo[*b*][1,4,7,11,15]dioxatriaza­cyclo­hepta­decine

**DOI:** 10.1107/S1600536810013097

**Published:** 2010-04-14

**Authors:** Zeynep Keleşoğlu, Elif Çelenk Kaya, Halit Kantekin, Orhan Büyükgüngör

**Affiliations:** a Department of Physics, Ondokuz Mayıs University, TR-55139, Samsun, Turkey; b Gümüşhane University, TR-29000, Gümüşhane, Turkey; c Department of Chemistry, Karadeniz Technical University, TR-61080, Trabzon, Turkey

## Abstract

In the title compound, C_31_H_39_Br_2_N_3_O_6_S_2_, a 17-membered aza-macrocyclic ligand containing two ether O and three aza N atoms, the three pendant aromatic rings form an ‘E’ shape. The dihedral angles between the central benzene ring and the side ones are 17.8 (3) and 7.4 (3)°, and the dihedral angle between the tosyl rings is 10.6 (3)°. The methyl group is disordered over two orientations, with occupancies of 0.52 (15) and 0.48 (15).

## Related literature

For general background to aza-macrocyclic ligands, see: Fry *et al.* (1997 ▶); Xu *et al.* (1997 ▶); Canales *et al.* (2000 ▶); Shishkina *et al.* (2007 ▶). For related structures, see: Hökelek *et al.* (2001 ▶, 2004 ▶); Işik *et al.* (1999 ▶). For further synthetic details, see: Notni *et al.* (2006 ▶); Koçak *et al.* (1994 ▶).
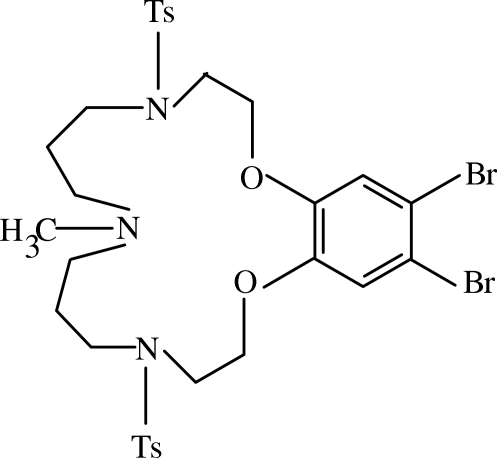


## Experimental

### 

#### Crystal data

C_31_H_39_Br_2_N_3_O_6_S_2_
*M* *_r_* = 773.59
Monoclinic, 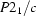

*a* = 18.7520 (9) Å
*b* = 10.6864 (4) Å
*c* = 19.9527 (9) Å
β = 121.416 (3)°
*V* = 3412.2 (3) Å^3^
*Z* = 4
Mo *K*α radiation
μ = 2.54 mm^−1^
*T* = 296 K
0.60 × 0.52 × 0.37 mm

#### Data collection

Stoe IPDS 2 diffractometer
Absorption correction: integration (*X-RED32*; Stoe & Cie, 2002 ▶) *T*_min_ = 0.260, *T*_max_ = 0.425
24321 measured reflections
7243 independent reflections
4640 reflections with *I* > 2σ(*I*)
*R*_int_ = 0.049

#### Refinement

*R*[*F* ^2^ > 2σ(*F* ^2^)] = 0.062
*wR*(*F* ^2^) = 0.152
*S* = 1.02
7243 reflections
409 parameters
H-atom parameters constrained
Δρ_max_ = 1.31 e Å^−3^
Δρ_min_ = −1.17 e Å^−3^

### 

Data collection: *X-AREA* (Stoe & Cie, 2002 ▶); cell refinement: *X-AREA*; data reduction: *X-RED32* (Stoe & Cie, 2002 ▶); program(s) used to solve structure: *SHELXS97* (Sheldrick, 2008 ▶); program(s) used to refine structure: *SHELXL97* (Sheldrick, 2008 ▶); molecular graphics: *ORTEP-3* (Farrugia, 1997 ▶); software used to prepare material for publication: *WinGX* (Farrugia, 1999 ▶).

## Supplementary Material

Crystal structure: contains datablocks I. DOI: 10.1107/S1600536810013097/hb5401sup1.cif

Structure factors: contains datablocks I. DOI: 10.1107/S1600536810013097/hb5401Isup2.hkl

Additional supplementary materials:  crystallographic information; 3D view; checkCIF report

## Figures and Tables

**Fig. 1.**
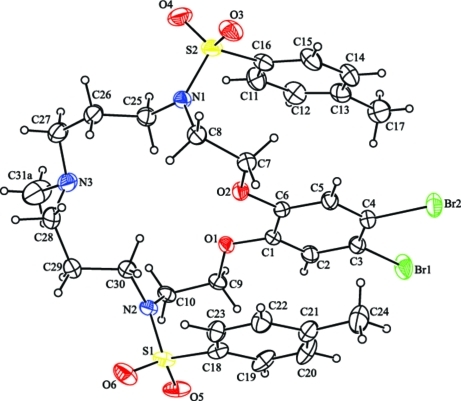
A view of (I), showing 30% probability displacement ellipsoids. Only the major disorder component of the methyl group is shown.

## supplementary crystallographic information

### Comment

The synthesis and characterization of coordination compounds with aza-macrocyclic ligands has evolved during the last years as one of the main research areas in coordination chemistry (Fry *et al.*,1997; Xu *et al.*, 1997). By the end of the last century the macrocyclic polyethers (crown ethers) had became one of the most popular chemical reagents with a very wide area of applications. They are used successfully in chemistry of 'host–guest' complexes, extraction, phase transfer catalysis, organic synthesis, analytical chemistry, biology, medicine, ecology, etc. (Shishkina *et al.*, 2007). In addition, aza-macrocyclic ligands, as well as their coordination and organometallic compounds play important roles in catalysis in the activation of small molecules, showing catalytic activity in electrochemically assisted reactions with several substrates (Canales *et al.*, 2000).

We have investigated the title structure of macrocyclic multidentate O_2_N_3_ donor-type ligand (Fig. 1). The 17-membered macrocyclic ring contains two ether O and three aza N atoms. The ligand cavity for macrocyclic ring plays an important role in metal-ion selectivity (Hökelek *et al.*,2004; Hökelek *et al.*, 2001).

The 17-membered macro-cyclic molecule with O_2_N_3_ type ring, the deviations from the least-squares plane defined by atoms O1, O2, N1, N2 and N3 are -0.661 (4)Å (O1), 0.363 (3)Å (O2), -0.352 (3)(N1) and 0.806 (3)Å (N2) and C29 shows the maximum r.m.s deviation from the plane as 1.067 (3).

The dihedral angle between the tosyl rings A(C18—C23, C24, S2) and B(C11—C16, C17, S1) is 10.6 (3)° [both nearly planar with r.m.s. deviations of 0.11 (3) Å for S1 and -0.06 (3) Å for S2, from the mean planes]. The geometry at the S atoms is distorted from the tetrahedral configuration [the largest angle is 120.3 (3)° for O3—S1—O4] and agree with the corresponding angle 120.4 (3)° in 10,11-Dibromo-3,6-ditosyl-3,6-diazabicyclo-[6.4.0]dodeca-1 (8),9,11-triene (Işik *et al.*, 1999).

The benzene rings C(C1—C6), D(C11—C16) and E(C18—C23) are planar with the maximum r.m.s. deviation from the mean plane as 0.021 (4) Å for C13. The dihedral angles between these benzene rings are C/D = 17.8 (3)°, D/E = 10.9 (3)° and C/E = 7.4 (3)°. The conformation of the title compound's macrocyclic ring can be given by the torsion angles. The optimum values of the torsion angles in a macrocyclic ring are 180° (anti) and 60° (gauche). In the compound (I), seven torsion angles are seems to be anti and five ones as gauche (Table 1). There is no classic hydrogen bonds in (I) and van der Waals interactions are effective in the molecular packing.

### Experimental

*N*,*N*'-(3,3'-(methylazanediyl)bis(propane-3,1-diyl))bis(4-methyl benzenesulfonamide) (Notni *et al.*, 2006) (1 g, 2.21 mmol) was dissolved in dry acetonitrile (50 ml) containing finely ground anhydrous Cs_2_CO_3_ (2.16 g, 6.63 mmol) and purged under nitrogen in a Schlenk system. This solution was stirred at 50 °C and a solution 1,2-bis(2-iodo-ethoxy) -4,5-dibromobenzene (Koçak *et al.*, 1994) (1.27 g, 2.21 mmol) in dry acetonitrile (30 ml) was added dropwise over a period of 3 h at reflux temperature (90 °C). The reaction was monitored by TLC using hexane/ethyl acetate (1:1) and was complete in 8 days at the reflux temperature. At the end of this period the solvent was removed under reduced pressure, mixed with water (50 ml) and then extracted with chloroform (3 times 50). The combine extract was washed with water, dried over Na_2_SO_4_ and filtered and evaporated to dryness. The product was chromatographed on silica gel with hexane/ethyl acetate (2:3). Finally the white solid product was obtained. This product was crystallized from chloroform/hexane to yield colourless prisms of (I).  This compound is soluble in chloroform, dichloromethane, dimethyl formamide. Yield: 0.65 g (%38). IR(KBr pellets): 3026 (Ar–H), 2926–2854 (Aliph. C–H), 1642, 1597, 1493, 1335, 1251, 1156, 815. 1H NMR (CDCl3): 7.67 (d, 4H, Ar–Ts—H), 7.22 (d, 4H, Ar–Ts—H), 6.81 (s, 2H, Ar–H), 3.97 (t, 4H, O–CH2), 3.66 (t, 4H, N–CH2), 3.45 (t, 4H, N–CH2), 2.37 (t, 4H, N–CH2), 2.21 (s, 6H, CH3), 2.08 (s, 3H, N—CH3), 1.70 (m, 4H, CH2). ^13^C NMR (CDCl3): 148.15 (Ar–C), 148.61 (Ar–C), 137.93 (Ar–C), 129.86 (Ar–C),127.06 (Ar–C), 117.47 (Ar–C), 115.25 (Ar–C), 69.21 (O–CH2), 54.26 (N–CH2), 48.58 (N–CH2), 47.96 (N–CH2), 41.56 (N–CH2), 26.98 (CH2), 21.77 (CH3).

### Refinement

All H atoms were positioned with idealized geometry using a riding model [C—H = 0.93—0.97 Å]. All H atoms were refined with isotropic displacement parameters (set to 1.2 and 1.5 times of the *U*_eq_ of the parent atom). The methyl group is disordered over two orientations, with occupancies of 0.52 (15) and O.48 (15).

### Crystal data

**Table:**

C_31_H_39_Br_2_N_3_O_6_S_2_	*F*(000) = 1584
*M**_r_* = 773.59	*D*_x_ = 1.506 Mg m^−^^3^
Monoclinic, *P*2_1_/*c*	Mo *K*α radiation, λ = 0.71073 Å
Hall symbol: -P 2ybc	Cell parameters from 22517 reflections
*a* = 18.7520 (9) Å	θ = 1.2–27.3°
*b* = 10.6864 (4) Å	µ = 2.54 mm^−^^1^
*c* = 19.9527 (9) Å	*T* = 296 K
β = 121.416 (3)°	Prism, colorless
*V* = 3412.2 (3) Å^3^	0.60 × 0.52 × 0.37 mm
*Z* = 4	

### Data collection

**Table:**

Stoe IPDS 2 diffractometer	7243 independent reflections
Radiation source: fine-focus sealed tube	4640 reflections with *I* > 2σ(*I*)
graphite	*R*_int_ = 0.049
Detector resolution: 6.67 pixels mm^-1^	θ_max_ = 26.8°, θ_min_ = 2.0°
rotation method scans	*h* = −23→21
Absorption correction: integration (*X-RED32*; Stoe & Cie, 2002)	*k* = −13→13
*T*_min_ = 0.260, *T*_max_ = 0.425	*l* = −25→25
24321 measured reflections	

### Refinement

**Table:**

Refinement on *F*^2^	Primary atom site location: structure-invariant direct methods
Least-squares matrix: full	Secondary atom site location: difference Fourier map
*R*[*F*^2^ > 2σ(*F*^2^)] = 0.062	Hydrogen site location: inferred from neighbouring sites
*wR*(*F*^2^) = 0.152	H-atom parameters constrained
*S* = 1.02	*w* = 1/[σ^2^(*F*_o_^2^) + (0.0629*P*)^2^ + 3.5929*P*] where *P* = (*F*_o_^2^ + 2*F*_c_^2^)/3
7243 reflections	(Δ/σ)_max_ = 0.001
409 parameters	Δρ_max_ = 1.31 e Å^−^^3^
0 restraints	Δρ_min_ = −1.17 e Å^−^^3^

### Special details

**Table:**

Geometry. All esds (except the esd in the dihedral angle between two l.s. planes) are estimated using the full covariance matrix. The cell esds are taken into account individually in the estimation of esds in distances, angles and torsion angles; correlations between esds in cell parameters are only used when they are defined by crystal symmetry. An approximate (isotropic) treatment of cell esds is used for estimating esds involving l.s. planes.
Refinement. Refinement of *F*^2^ against ALL reflections. The weighted *R*-factor wR and goodness of fit *S* are based on *F*^2^, conventional *R*-factors *R* are based on *F*, with *F* set to zero for negative *F*^2^. The threshold expression of *F*^2^ > σ(*F*^2^) is used only for calculating *R*-factors(gt) etc. and is not relevant to the choice of reflections for refinement. *R*-factors based on *F*^2^ are statistically about twice as large as those based on *F*, and *R*- factors based on ALL data will be even larger.

### Fractional atomic coordinates and isotropic or equivalent isotropic displacement parameters (Å^2^)

**Table:**

	*x*	*y*	*z*	*U*_iso_*/*U*_eq_	Occ. (<1)
C1	0.7001 (3)	0.3914 (4)	0.5560 (2)	0.0486 (10)	
C2	0.6569 (3)	0.2996 (4)	0.5022 (3)	0.0632 (12)	
H2	0.6427	0.2263	0.5176	0.076*	
C3	0.6341 (3)	0.3154 (4)	0.4237 (3)	0.0655 (13)	
C4	0.6564 (3)	0.4214 (5)	0.4013 (2)	0.0585 (11)	
C5	0.6994 (3)	0.5155 (4)	0.4557 (2)	0.0556 (11)	
H5	0.7138	0.5883	0.4400	0.067*	
C6	0.7208 (3)	0.5021 (4)	0.5325 (2)	0.0468 (9)	
C7	0.7871 (3)	0.7025 (4)	0.5702 (3)	0.0573 (11)	
H7A	0.7425	0.7350	0.5203	0.069*	
H7B	0.8351	0.6855	0.5656	0.069*	
C8	0.8097 (3)	0.7969 (4)	0.6346 (3)	0.0579 (11)	
H8A	0.8537	0.7617	0.6838	0.069*	
H8B	0.8322	0.8707	0.6238	0.069*	
C9	0.7245 (3)	0.2609 (4)	0.6628 (3)	0.0564 (11)	
H9A	0.7463	0.2000	0.6419	0.068*	
H9B	0.6681	0.2367	0.6474	0.068*	
C10	0.7790 (3)	0.2666 (5)	0.7508 (2)	0.0569 (11)	
H10A	0.7519	0.3193	0.7706	0.068*	
H10B	0.7832	0.1831	0.7716	0.068*	
C11	0.5504 (3)	0.7378 (6)	0.5204 (3)	0.0806 (15)	
H11	0.5552	0.7363	0.5692	0.097*	
C12	0.4984 (3)	0.6531 (6)	0.4624 (4)	0.0842 (17)	
H12	0.4678	0.5964	0.4729	0.101*	
C13	0.4909 (3)	0.6504 (5)	0.3900 (3)	0.0723 (14)	
C14	0.5329 (3)	0.7394 (6)	0.3749 (3)	0.0813 (16)	
H14	0.5267	0.7422	0.3256	0.098*	
C15	0.5846 (3)	0.8260 (5)	0.4315 (3)	0.0790 (16)	
H15	0.6126	0.8857	0.4199	0.095*	
C16	0.5944 (3)	0.8236 (5)	0.5051 (3)	0.0645 (12)	
C17	0.4383 (4)	0.5511 (6)	0.3308 (4)	0.098 (2)	
H17A	0.3818	0.5570	0.3189	0.118*	
H17B	0.4603	0.4699	0.3521	0.118*	
H17C	0.4394	0.5633	0.2836	0.118*	
C18	0.9238 (3)	0.2680 (4)	0.6840 (3)	0.0580 (11)	
C19	0.8771 (4)	0.1928 (5)	0.6181 (4)	0.0787 (16)	
H19	0.8517	0.1203	0.6215	0.094*	
C20	0.8690 (4)	0.2273 (6)	0.5484 (4)	0.0888 (19)	
H20	0.8380	0.1763	0.5048	0.107*	
C21	0.9045 (3)	0.3334 (5)	0.5398 (3)	0.0692 (13)	
C22	0.9515 (3)	0.4052 (5)	0.6057 (3)	0.0694 (13)	
H22	0.9773	0.4770	0.6021	0.083*	
C23	0.9614 (3)	0.3740 (5)	0.6772 (3)	0.0631 (12)	
H23	0.9934	0.4245	0.7208	0.076*	
C24	0.8933 (5)	0.3707 (7)	0.4621 (4)	0.106 (2)	
H24A	0.9469	0.3748	0.4669	0.128*	
H24B	0.8668	0.4511	0.4470	0.128*	
H24C	0.8590	0.3098	0.4230	0.128*	
C25	0.7356 (3)	0.7777 (5)	0.7079 (3)	0.0642 (12)	
H25A	0.7467	0.6888	0.7092	0.077*	
H25B	0.6793	0.7876	0.6975	0.077*	
C26	0.7965 (4)	0.8339 (5)	0.7868 (3)	0.0759 (15)	
H26A	0.7847	0.9225	0.7857	0.091*	
H26B	0.8527	0.8256	0.7966	0.091*	
C27	0.7927 (5)	0.7729 (5)	0.8533 (3)	0.0908 (19)	
H27A	0.8327	0.8134	0.9022	0.109*	
H27B	0.7374	0.7858	0.8450	0.109*	
C28	0.8987 (4)	0.6155 (6)	0.8929 (3)	0.101 (2)	
H28A	0.9289	0.6346	0.9486	0.122*	
H28B	0.9193	0.6707	0.8681	0.122*	
C29	0.9159 (4)	0.4810 (6)	0.8816 (3)	0.094 (2)	
H29A	0.9758	0.4678	0.9086	0.113*	
H29B	0.8937	0.4256	0.9048	0.113*	
C30	0.8775 (3)	0.4486 (4)	0.7963 (3)	0.0644 (12)	
H30A	0.8245	0.4920	0.7662	0.077*	
H30B	0.9138	0.4782	0.7786	0.077*	
C31A	0.797 (8)	0.589 (5)	0.919 (6)	0.14 (2)	0.52 (15)
H31A	0.8063	0.4999	0.9222	0.205*	0.52 (15)
H31B	0.7410	0.6057	0.9052	0.205*	0.52 (15)
H31C	0.8357	0.6266	0.9684	0.205*	0.52 (15)
C31B	0.758 (5)	0.570 (6)	0.890 (4)	0.118 (12)	0.48 (15)
H31D	0.7627	0.4809	0.8864	0.178*	0.48 (15)
H31E	0.7007	0.5943	0.8591	0.178*	0.48 (15)
H31F	0.7797	0.5918	0.9443	0.178*	0.48 (15)
N1	0.7410 (2)	0.8354 (3)	0.6445 (2)	0.0543 (9)	
N2	0.8634 (2)	0.3142 (3)	0.7806 (2)	0.0545 (9)	
N3	0.8103 (4)	0.6388 (4)	0.8600 (3)	0.0798 (13)	
O1	0.72458 (19)	0.3840 (3)	0.63322 (15)	0.0562 (7)	
O2	0.76047 (19)	0.5906 (3)	0.58982 (16)	0.0551 (7)	
O3	0.7025 (3)	0.9994 (3)	0.5462 (2)	0.0861 (12)	
O4	0.6274 (3)	0.9787 (4)	0.6179 (3)	0.0931 (12)	
O5	0.9052 (3)	0.1027 (3)	0.7685 (3)	0.0910 (12)	
O6	1.0111 (2)	0.2702 (4)	0.8347 (2)	0.0906 (12)	
S1	0.66723 (9)	0.92262 (11)	0.58044 (8)	0.0680 (3)	
S2	0.93076 (8)	0.23094 (12)	0.77324 (8)	0.0643 (3)	
Br1	0.56928 (5)	0.18578 (6)	0.35290 (3)	0.1104 (3)	
Br2	0.62827 (4)	0.44795 (6)	0.29617 (3)	0.0819 (2)	

### Atomic displacement parameters (Å^2^)

**Table:**

	*U*^11^	*U*^22^	*U*^33^	*U*^12^	*U*^13^	*U*^23^
C1	0.050 (2)	0.046 (2)	0.043 (2)	0.0069 (19)	0.0199 (18)	0.0034 (17)
C2	0.079 (3)	0.044 (2)	0.055 (2)	−0.001 (2)	0.026 (2)	0.0036 (19)
C3	0.077 (3)	0.051 (3)	0.050 (2)	0.002 (2)	0.021 (2)	−0.004 (2)
C4	0.063 (3)	0.064 (3)	0.045 (2)	0.005 (2)	0.026 (2)	0.001 (2)
C5	0.063 (3)	0.053 (3)	0.049 (2)	−0.002 (2)	0.028 (2)	0.0026 (19)
C6	0.048 (2)	0.044 (2)	0.046 (2)	0.0026 (18)	0.0217 (18)	−0.0010 (17)
C7	0.060 (3)	0.052 (3)	0.060 (2)	−0.008 (2)	0.031 (2)	0.001 (2)
C8	0.057 (3)	0.051 (3)	0.060 (3)	−0.010 (2)	0.026 (2)	−0.001 (2)
C9	0.059 (3)	0.047 (2)	0.059 (2)	0.004 (2)	0.028 (2)	0.012 (2)
C10	0.057 (3)	0.062 (3)	0.056 (2)	0.012 (2)	0.032 (2)	0.020 (2)
C11	0.068 (3)	0.096 (4)	0.069 (3)	−0.011 (3)	0.030 (3)	0.004 (3)
C12	0.058 (3)	0.093 (4)	0.095 (4)	−0.017 (3)	0.035 (3)	0.004 (3)
C13	0.045 (3)	0.078 (4)	0.072 (3)	0.002 (2)	0.016 (2)	0.003 (3)
C14	0.070 (3)	0.094 (4)	0.057 (3)	0.000 (3)	0.018 (3)	0.011 (3)
C15	0.073 (4)	0.074 (3)	0.067 (3)	−0.010 (3)	0.020 (3)	0.021 (3)
C16	0.056 (3)	0.060 (3)	0.065 (3)	0.010 (2)	0.022 (2)	0.014 (2)
C17	0.068 (4)	0.103 (5)	0.100 (4)	−0.011 (3)	0.027 (3)	−0.015 (4)
C18	0.055 (3)	0.048 (2)	0.078 (3)	0.003 (2)	0.039 (2)	−0.005 (2)
C19	0.095 (4)	0.053 (3)	0.117 (5)	−0.020 (3)	0.075 (4)	−0.031 (3)
C20	0.099 (4)	0.088 (4)	0.095 (4)	−0.034 (4)	0.062 (4)	−0.054 (3)
C21	0.070 (3)	0.074 (3)	0.071 (3)	−0.004 (3)	0.042 (3)	−0.018 (3)
C22	0.083 (4)	0.061 (3)	0.073 (3)	−0.013 (3)	0.046 (3)	−0.007 (2)
C23	0.065 (3)	0.060 (3)	0.063 (3)	−0.014 (2)	0.032 (2)	−0.012 (2)
C24	0.127 (6)	0.128 (6)	0.073 (4)	0.000 (5)	0.058 (4)	−0.016 (4)
C25	0.071 (3)	0.058 (3)	0.063 (3)	−0.001 (2)	0.035 (3)	0.005 (2)
C26	0.105 (4)	0.058 (3)	0.066 (3)	0.004 (3)	0.045 (3)	−0.003 (2)
C27	0.142 (6)	0.070 (4)	0.073 (3)	0.020 (4)	0.065 (4)	0.003 (3)
C28	0.117 (6)	0.080 (4)	0.059 (3)	0.009 (4)	0.013 (3)	−0.016 (3)
C29	0.100 (4)	0.082 (4)	0.056 (3)	0.022 (3)	0.010 (3)	−0.005 (3)
C30	0.076 (3)	0.057 (3)	0.057 (3)	0.010 (2)	0.033 (2)	0.006 (2)
C31A	0.23 (6)	0.092 (18)	0.16 (4)	0.02 (3)	0.15 (4)	0.03 (2)
C31B	0.18 (3)	0.12 (2)	0.10 (2)	−0.02 (2)	0.11 (2)	0.002 (16)
N1	0.055 (2)	0.049 (2)	0.0530 (19)	0.0003 (17)	0.0237 (17)	0.0008 (16)
N2	0.054 (2)	0.052 (2)	0.057 (2)	0.0107 (17)	0.0285 (18)	0.0106 (16)
N3	0.121 (4)	0.067 (3)	0.068 (3)	0.017 (3)	0.060 (3)	0.009 (2)
O1	0.071 (2)	0.0450 (16)	0.0431 (14)	0.0023 (14)	0.0232 (14)	0.0057 (12)
O2	0.0665 (19)	0.0474 (16)	0.0483 (15)	−0.0081 (14)	0.0277 (14)	−0.0018 (13)
O3	0.098 (3)	0.0517 (19)	0.084 (2)	−0.0102 (19)	0.030 (2)	0.0179 (18)
O4	0.089 (3)	0.072 (2)	0.113 (3)	0.022 (2)	0.049 (2)	−0.010 (2)
O5	0.104 (3)	0.0483 (19)	0.143 (4)	0.023 (2)	0.080 (3)	0.030 (2)
O6	0.055 (2)	0.121 (3)	0.082 (2)	0.018 (2)	0.0259 (19)	0.027 (2)
S1	0.0714 (8)	0.0436 (6)	0.0742 (8)	0.0055 (6)	0.0275 (7)	0.0053 (5)
S2	0.0548 (7)	0.0589 (7)	0.0799 (8)	0.0187 (6)	0.0355 (6)	0.0218 (6)
Br1	0.1618 (7)	0.0672 (4)	0.0607 (3)	−0.0270 (4)	0.0290 (4)	−0.0158 (3)
Br2	0.1014 (5)	0.0923 (4)	0.0486 (3)	−0.0089 (3)	0.0368 (3)	−0.0056 (3)

### Geometric parameters (Å, °)

**Table:**

C1—O1	1.361 (5)	C19—H19	0.9300
C1—C2	1.366 (6)	C20—C21	1.370 (8)
C1—C6	1.400 (6)	C20—H20	0.9300
C2—C3	1.401 (6)	C21—C22	1.374 (7)
C2—H2	0.9300	C21—C24	1.504 (8)
C3—C4	1.361 (7)	C22—C23	1.379 (7)
C3—Br1	1.898 (5)	C22—H22	0.9300
C4—C5	1.388 (6)	C23—H23	0.9300
C4—Br2	1.899 (4)	C24—H24A	0.9600
C5—C6	1.374 (5)	C24—H24B	0.9600
C5—H5	0.9300	C24—H24C	0.9600
C6—O2	1.368 (5)	C25—N1	1.456 (6)
C7—O2	1.427 (5)	C25—C26	1.506 (7)
C7—C8	1.510 (6)	C25—H25A	0.9700
C7—H7A	0.9700	C25—H25B	0.9700
C7—H7B	0.9700	C26—C27	1.513 (7)
C8—N1	1.462 (6)	C26—H26A	0.9700
C8—H8A	0.9700	C26—H26B	0.9700
C8—H8B	0.9700	C27—N3	1.462 (7)
C9—O1	1.442 (5)	C27—H27A	0.9700
C9—C10	1.503 (6)	C27—H27B	0.9700
C9—H9A	0.9700	C28—N3	1.451 (8)
C9—H9B	0.9700	C28—C29	1.516 (8)
C10—N2	1.461 (6)	C28—H28A	0.9700
C10—H10A	0.9700	C28—H28B	0.9700
C10—H10B	0.9700	C29—C30	1.501 (7)
C11—C16	1.371 (7)	C29—H29A	0.9700
C11—C12	1.390 (8)	C29—H29B	0.9700
C11—H11	0.9300	C30—N2	1.465 (6)
C12—C13	1.376 (8)	C30—H30A	0.9700
C12—H12	0.9300	C30—H30B	0.9700
C13—C14	1.365 (8)	C31A—N3	1.42 (4)
C13—C17	1.511 (8)	C31A—H31A	0.9600
C14—C15	1.389 (8)	C31A—H31B	0.9600
C14—H14	0.9300	C31A—H31C	0.9600
C15—C16	1.380 (7)	C31B—N3	1.58 (6)
C15—H15	0.9300	C31B—H31D	0.9600
C16—S1	1.762 (5)	C31B—H31E	0.9600
C17—H17A	0.9600	C31B—H31F	0.9600
C17—H17B	0.9600	N1—S1	1.604 (4)
C17—H17C	0.9600	N2—S2	1.613 (4)
C18—C23	1.377 (7)	O3—S1	1.432 (4)
C18—C19	1.392 (7)	O4—S1	1.434 (4)
C18—S2	1.762 (5)	O5—S2	1.439 (4)
C19—C20	1.370 (8)	O6—S2	1.423 (4)
			
O1—C1—C2	124.1 (4)	C23—C22—H22	119.1
O1—C1—C6	116.1 (4)	C18—C23—C22	120.0 (5)
C2—C1—C6	119.8 (4)	C18—C23—H23	120.0
C1—C2—C3	120.2 (4)	C22—C23—H23	120.0
C1—C2—H2	119.9	C21—C24—H24A	109.5
C3—C2—H2	119.9	C21—C24—H24B	109.5
C4—C3—C2	120.0 (4)	H24A—C24—H24B	109.5
C4—C3—Br1	123.4 (3)	C21—C24—H24C	109.5
C2—C3—Br1	116.6 (4)	H24A—C24—H24C	109.5
C3—C4—C5	120.0 (4)	H24B—C24—H24C	109.5
C3—C4—Br2	122.1 (3)	N1—C25—C26	112.4 (4)
C5—C4—Br2	117.9 (4)	N1—C25—H25A	109.1
C6—C5—C4	120.6 (4)	C26—C25—H25A	109.1
C6—C5—H5	119.7	N1—C25—H25B	109.1
C4—C5—H5	119.7	C26—C25—H25B	109.1
O2—C6—C5	125.2 (4)	H25A—C25—H25B	107.9
O2—C6—C1	115.4 (3)	C25—C26—C27	112.8 (5)
C5—C6—C1	119.4 (4)	C25—C26—H26A	109.0
O2—C7—C8	108.0 (3)	C27—C26—H26A	109.0
O2—C7—H7A	110.1	C25—C26—H26B	109.0
C8—C7—H7A	110.1	C27—C26—H26B	109.0
O2—C7—H7B	110.1	H26A—C26—H26B	107.8
C8—C7—H7B	110.1	N3—C27—C26	113.0 (4)
H7A—C7—H7B	108.4	N3—C27—H27A	109.0
N1—C8—C7	115.3 (4)	C26—C27—H27A	109.0
N1—C8—H8A	108.5	N3—C27—H27B	109.0
C7—C8—H8A	108.5	C26—C27—H27B	109.0
N1—C8—H8B	108.5	H27A—C27—H27B	107.8
C7—C8—H8B	108.5	N3—C28—C29	112.1 (5)
H8A—C8—H8B	107.5	N3—C28—H28A	109.2
O1—C9—C10	107.2 (4)	C29—C28—H28A	109.2
O1—C9—H9A	110.3	N3—C28—H28B	109.2
C10—C9—H9A	110.3	C29—C28—H28B	109.2
O1—C9—H9B	110.3	H28A—C28—H28B	107.9
C10—C9—H9B	110.3	C30—C29—C28	112.0 (5)
H9A—C9—H9B	108.5	C30—C29—H29A	109.2
N2—C10—C9	115.0 (4)	C28—C29—H29A	109.2
N2—C10—H10A	108.5	C30—C29—H29B	109.2
C9—C10—H10A	108.5	C28—C29—H29B	109.2
N2—C10—H10B	108.5	H29A—C29—H29B	107.9
C9—C10—H10B	108.5	N2—C30—C29	113.3 (4)
H10A—C10—H10B	107.5	N2—C30—H30A	108.9
C16—C11—C12	119.5 (5)	C29—C30—H30A	108.9
C16—C11—H11	120.2	N2—C30—H30B	108.9
C12—C11—H11	120.2	C29—C30—H30B	108.9
C13—C12—C11	122.0 (6)	H30A—C30—H30B	107.7
C13—C12—H12	119.0	N3—C31A—H31A	109.5
C11—C12—H12	119.0	N3—C31A—H31B	109.5
C14—C13—C12	117.6 (5)	N3—C31A—H31C	109.5
C14—C13—C17	122.2 (6)	N3—C31B—H31D	109.5
C12—C13—C17	120.2 (6)	N3—C31B—H31E	109.5
C13—C14—C15	121.5 (5)	H31D—C31B—H31E	109.5
C13—C14—H14	119.3	N3—C31B—H31F	109.5
C15—C14—H14	119.3	H31D—C31B—H31F	109.5
C16—C15—C14	120.2 (5)	H31E—C31B—H31F	109.5
C16—C15—H15	119.9	C25—N1—C8	117.6 (4)
C14—C15—H15	119.9	C25—N1—S1	121.2 (3)
C11—C16—C15	119.1 (5)	C8—N1—S1	120.7 (3)
C11—C16—S1	120.0 (4)	C10—N2—C30	117.7 (4)
C15—C16—S1	120.7 (4)	C10—N2—S2	120.3 (3)
C13—C17—H17A	109.5	C30—N2—S2	119.9 (3)
C13—C17—H17B	109.5	C31A—N3—C28	102 (5)
H17A—C17—H17B	109.5	C31A—N3—C27	109 (2)
C13—C17—H17C	109.5	C28—N3—C27	111.2 (5)
H17A—C17—H17C	109.5	C28—N3—C31B	122 (3)
H17B—C17—H17C	109.5	C27—N3—C31B	110 (2)
C23—C18—C19	119.0 (5)	C1—O1—C9	116.4 (3)
C23—C18—S2	120.8 (4)	C6—O2—C7	117.8 (3)
C19—C18—S2	120.2 (4)	O3—S1—O4	120.3 (3)
C20—C19—C18	119.0 (5)	O3—S1—N1	107.0 (2)
C20—C19—H19	120.5	O4—S1—N1	107.1 (2)
C18—C19—H19	120.5	O3—S1—C16	106.4 (2)
C21—C20—C19	123.1 (5)	O4—S1—C16	108.2 (3)
C21—C20—H20	118.4	N1—S1—C16	107.1 (2)
C19—C20—H20	118.4	O6—S2—O5	120.3 (3)
C20—C21—C22	116.9 (5)	O6—S2—N2	106.7 (2)
C20—C21—C24	122.1 (5)	O5—S2—N2	106.3 (2)
C22—C21—C24	120.9 (5)	O6—S2—C18	106.9 (2)
C21—C22—C23	121.9 (5)	O5—S2—C18	107.5 (2)
C21—C22—H22	119.1	N2—S2—C18	108.7 (2)
			
O1—C1—C2—C3	179.8 (4)	C26—C25—N1—S1	−110.2 (4)
C6—C1—C2—C3	0.6 (7)	C7—C8—N1—C25	100.3 (5)
C1—C2—C3—C4	1.4 (8)	C7—C8—N1—S1	−71.1 (5)
C1—C2—C3—Br1	−176.8 (4)	C9—C10—N2—C30	92.1 (5)
C2—C3—C4—C5	−2.2 (8)	C9—C10—N2—S2	−71.1 (5)
Br1—C3—C4—C5	175.9 (4)	C29—C30—N2—C10	99.7 (5)
C2—C3—C4—Br2	179.4 (4)	C29—C30—N2—S2	−97.0 (5)
Br1—C3—C4—Br2	−2.5 (6)	C29—C28—N3—C31A	79 (4)
C3—C4—C5—C6	0.9 (7)	C29—C28—N3—C27	−165.4 (5)
Br2—C4—C5—C6	179.4 (3)	C29—C28—N3—C31B	62 (3)
C4—C5—C6—O2	−177.6 (4)	C26—C27—N3—C31A	−174 (6)
C4—C5—C6—C1	1.1 (7)	C26—C27—N3—C28	74.0 (7)
O1—C1—C6—O2	−2.3 (5)	C26—C27—N3—C31B	−147 (3)
C2—C1—C6—O2	177.0 (4)	C2—C1—O1—C9	17.1 (6)
O1—C1—C6—C5	178.9 (4)	C6—C1—O1—C9	−163.6 (4)
C2—C1—C6—C5	−1.8 (6)	C10—C9—O1—C1	161.3 (4)
O2—C7—C8—N1	−62.9 (5)	C5—C6—O2—C7	−4.6 (6)
O1—C9—C10—N2	−54.1 (5)	C1—C6—O2—C7	176.7 (4)
C16—C11—C12—C13	1.4 (9)	C8—C7—O2—C6	166.7 (4)
C11—C12—C13—C14	−3.7 (9)	C25—N1—S1—O3	159.0 (3)
C11—C12—C13—C17	175.8 (6)	C8—N1—S1—O3	−30.0 (4)
C12—C13—C14—C15	3.1 (9)	C25—N1—S1—O4	28.7 (4)
C17—C13—C14—C15	−176.5 (5)	C8—N1—S1—O4	−160.3 (3)
C13—C14—C15—C16	−0.1 (9)	C25—N1—S1—C16	−87.3 (4)
C12—C11—C16—C15	1.6 (8)	C8—N1—S1—C16	83.8 (4)
C12—C11—C16—S1	−174.5 (4)	C11—C16—S1—O3	179.6 (4)
C14—C15—C16—C11	−2.2 (8)	C15—C16—S1—O3	3.5 (5)
C14—C15—C16—S1	173.8 (4)	C11—C16—S1—O4	−49.8 (5)
C23—C18—C19—C20	−0.7 (8)	C15—C16—S1—O4	134.2 (5)
S2—C18—C19—C20	176.5 (4)	C11—C16—S1—N1	65.4 (5)
C18—C19—C20—C21	−0.4 (9)	C15—C16—S1—N1	−110.6 (4)
C19—C20—C21—C22	1.4 (9)	C10—N2—S2—O6	−154.2 (3)
C19—C20—C21—C24	−178.7 (6)	C30—N2—S2—O6	42.9 (4)
C20—C21—C22—C23	−1.2 (8)	C10—N2—S2—O5	−24.7 (4)
C24—C21—C22—C23	178.8 (5)	C30—N2—S2—O5	172.5 (3)
C19—C18—C23—C22	0.9 (8)	C10—N2—S2—C18	90.8 (3)
S2—C18—C23—C22	−176.4 (4)	C30—N2—S2—C18	−72.0 (4)
C21—C22—C23—C18	0.1 (8)	C23—C18—S2—O6	−35.0 (5)
N1—C25—C26—C27	−178.9 (4)	C19—C18—S2—O6	147.8 (4)
C25—C26—C27—N3	59.2 (7)	C23—C18—S2—O5	−165.5 (4)
N3—C28—C29—C30	64.1 (8)	C19—C18—S2—O5	17.3 (5)
C28—C29—C30—N2	−157.2 (6)	C23—C18—S2—N2	79.8 (4)
C26—C25—N1—C8	78.5 (5)	C19—C18—S2—N2	−97.4 (4)
